# Knowledge and Educational Needs about Pre-Implantation Genetic Diagnosis (PGD) among Oncology Nurses

**DOI:** 10.3390/jcm3020632

**Published:** 2014-06-20

**Authors:** Gwendolyn P. Quinn, Caprice Knapp, Ivana Sehovic, Danielle Ung, Meghan Bowman, Luis Gonzalez, Susan T. Vadaparampil

**Affiliations:** 1 Department of Oncologic Sciences, Morsani College of Medicine, University of South Florida, 12901 Bruce B Downs Blvd #11, Tampa, FL 33612, USA; E-Mail: susan.vadaparampil@moffitt.org; 2 Department of Health Outcomes & Behavior, H. Lee Moffitt Cancer Center and Research Institute, Moffitt Cancer Center, MRC CANCONT, 12902 Magnolia Drive, Tampa, FL 33612, USA; E-Mails: ivana.sehovic@moffitt.org (I.S.); danielle.ung@moffitt.org (D.U.); meghan.bowman@moffitt.org (M.B.); lgonzal3@health.usf.edu (L.G.); 3 Department of Health Outcomes and Policy, University of Florida, 1329 SW 16th St., Gainesville, FL 32608, USA; E-Mail: caprice1@ufl.edu

**Keywords:** nurse, pre implantation genetic diagnosis, oncology, hereditary cancer

## Abstract

Preimplantation genetic diagnosis (PGD), a form of assisted reproductive technology, is a new technology with limited awareness among health care professionals and hereditary cancer families. Nurses play a key role in the care of patients and are often in an ideal position to discuss and refer patients on sensitive quality of life issues, such as PGD. Two hundred and one nurses at Moffitt Cancer Center (MCC) responded to an online survey assessing knowledge and educational needs regarding PGD and families with hereditary cancer. The majority of respondents were female (*n* = 188), white (*n* = 175), had an RN/BSN degree (*n* = 83), and provided outpatient care at the cancer center (*n* = 102). More than half of respondents (78%) were unfamiliar with PGD prior to the survey and respondents who had heard of PGD had limited knowledge. More than half of the participants reported PGD was an acceptable option for families with hereditary cancer syndromes and thought individuals with a strong family or personal history should be provided with information about PGD. This study indicates that oncology nurses may benefit from and desire education about PGD. With advances in reproductive technology and options, further PGD education is needed among healthcare professionals. An examination of current oncology nursing curriculum and competencies regarding genetic education may identify need for future revisions and updates.

## 1. Introduction

### 1.1. Background on PGD

Preimplantation genetic diagnosis (PGD), a form of assisted reproductive technology (ART), is a medical procedure that allows couples to choose which fertilized embryos, created through *in vitro* fertilization (IVF) and tested for genetic disorders, are implanted into a woman’s uterus for further gestation, with the intent that the pregnancy will result in a healthy child, free of the tested genetic diseases [1]. PGD was first pioneered in 1968 and was successfully used in the 1990s as an ART tool for parents who feared that they would pass on a serious and/or life threatening genetic condition to their children such as cystic fibrosis, Fragile X syndrome, Down syndrome, Huntington’s disease, and sickle-cell anemia [1,2,3]. Since then, PGD has been used to identify over 200 conditions, and over 20,000 cases of PGD use have been reported in the U.S. [4]. PGD may be beneficial for individuals who have a family history of cancer and are concerned about passing on this genetic risk to their offspring [5]. PGD has been used to identify cancer predispositions in 22 common inherited cancer syndromes including breast cancer (*BRCA1* and *BRCA2*), Gorlin syndrome, tuberous sclerosis, familial colorectal cancer, and retinoblastoma [6,7].

As genetic counseling and testing are becoming regularly used by individuals who are affected by or at risk for hereditary diseases, a growing number of women are considering the implications of this risk on childbearing decisions [8]. PGD provides a safer alternative to prenatal diagnoses and in some cases, has been reported to be less stressful [9]. Prenatal testing such as amniocentesis and CVS is often described as an invasive and risky procedure (e.g., risk of miscarriage) and may lead to a decision regarding pregnancy termination, as the fetus is tested for genetic disorders while in utero. In comparison, PGD offers the option for testing *in vitro* embryos and implanting those without the genetic mutation of concern. Additionally, IVF embryo creation offers other benefits such as significantly improved implantation, lower spontaneous abortion rates and more successful and healthier births [10,11,12].

### 1.2. Patient and Family Attitudes toward PGD

Individuals who are at increased genetic risk for cancer are often worried about their chances of passing the disease to their offspring, which can significantly affect their reproduction decisions [13]. The factors affecting their beliefs and attitudes towards reproductive genetic technologies (RGTs) are complex. For example, Kalfoglou, *et al.* [14] found that six key factors determined the appropriateness of RGTs in assisting with reproduction. These factors included whether the embryos would be destroyed, the nature of the disease or trait being avoided or sought, technological control over “natural” reproduction, the value of suffering, disability, and diversity, the importance of having genetically related children, and the type of future people desire or fear. Furthermore, genetic and reproductive history such as type of genetic condition, past pregnancy terminations, and previous miscarriages may impact the acceptability of PGD over other methods [15].

Due to the complex issues surrounding PGD, patient and family attitudes toward PGD have been mixed, with participants in some studies reporting that they would not consider using PGD [16] while other studies reported more receptive responses [17,18], especially in participants who carry a familial genetic risk such as cancer or have been diagnosed with cancer [19]. Interestingly, some women were in favor of PGD for others but would not consider it themselves [20]. In a recent study, Quinn, Vadaparampil, Wilson, King, Choi, Miree and Friedman [16] found that the main perceived worry about PGD among participants was that it could be used for the wrong purposes, “too much like playing God”, and that most people would not be able to afford it. These participants also reported that the main perceived benefit of PGD was an improved chance that the child would be free of the familial genetic mutation and that certain genetic diseases could be wiped out forever. That study highlights the conflicting opinions that consumers may have when considering PGD. 

The medical process of PGD has been described as a stressful and emotional experience by women, fraught with feelings of hope, disappointment, anxiety, and depression which may result from having to endure multiple embryo transfers, IVF cycles, and pregnancy testing [9,21,22]. However, many women with a high risk of hereditary cancer have expressed their perceptions that PGD is an acceptable option for themselves as well as other high-risk individuals, and that it is good ethical practice to inform women/couples of this option [16,19]. PGD has been viewed as more ethically acceptable than prenatal diagnoses by some users and their families because the genetic testing and manipulation of the embryo occurs outside of the uterus and an embryo that is not implanted is usually not considered to be a terminated pregnancy, which can result in physical and/or emotional trauma for the families [23,24,25].

Consumers have identified several advantages and disadvantages associated with PGD [15,26]. Advantages include reduced risk of passing on a genetic disease to offspring, reducing the chance of pregnancy terminations and miscarriages, and avoidance of emotional stress associated with waiting to know or having a child with a genetic disease. Disadvantages include moral dilemmas surrounding the idea of a “designer baby” or what to do with additional embryos, risk of misdiagnosis, costs, potential damage to the embryo, failure to conceive, and the physical and emotional burdens of IVF. A systematic and narrative literature review of PGD revealed that although patients and their families generally have positive attitudes toward PGD, their views of PGD for themselves and others are often conflicting, especially regarding their intentions and their actual decision to use PGD [15,27].

### 1.3. Nurses Involvement with PGD

Often a controversial topic of discussion, PGD has invoked varying opinions among health care professionals, potential consumers, and families [28]. Findings indicate PGD is generally accepted among professionals to avoid life-threatening genetic diseases but not to select for socially desirable traits or non-medical sex selection [26,28,29]. While studies of physicians and genetic counselors’ attitudes towards PGD have been explored [30,31], there has been little research on nurses’ knowledge and educational needs regarding this topic. Nurses are in an ideal position to inform patients and consumers about health-related quality of life options and provide information that aids in health-related decision-making. 

Nurses may be involved with the PGD process by relaying PGD information to patients and providing support to patients who are interested and/or involved in these procedures. Consequently, their opinions and beliefs about PGD may affect what and how much information they give to their patients, which may in turn affect their patients’ reproductive decisions. 

### 1.4. Importance of Studying Nurse Knowledge and Needs for Education

As many as 85% of healthcare professionals are involved in providing information and clinical care about infertility issues [29]. As nurses play a key role in the care of patients and have multiple interactions with patients, they are in an ideal position to discuss and refer patients on sensitive quality of life issues, such as PGD. They may be the first source of contact regarding reproductive options. Their experience and expertise may help to improve clinical procedures and policies and stimulate discussions in regards to the presentation and dissemination of PGD information [26]. Despite nurses’ ideal position to assist with important quality of life decisions, few studies have examined their knowledge and attitudes about PGD.

### 1.5. Study Aims

This primary aims of the study were to: (1) identify factors related to nurses’ awareness of PGD; (2) determine barriers that were associated with awareness of PGD; and (3) identify nurses’ informational preferences regarding PGD.

## 2. Experimental Section

### 2.1. Participants and Recruitment Procedures

The chief nursing officer at the Cancer Center provided approval for use of RN email addresses for participation in the survey. Study approval (project MCC 15191) was obtained from the University of South Florida (USF) Institutional Review Board (IRB) on 13 April 2007. An email was sent to all nurses on the Cancer Center email listserv with an invitation and link to access the web survey. Participation was voluntary and anonymous. The survey was available for 6 weeks. 

### 2.2. Measures

A modified survey originally created for a patient/consumer population on PGD was administered [32]. The survey consisted of 32 items divided into three sections intended to measure demographics; knowledge and awareness; and educational needs regarding PGD among oncology nurses at the cancer center. Nurses were asked to indicate if they were aware of a genetic counseling program at the cancer center, if they ever personally had a cancer-related genetic test, and if they or their spouse/partner ever tested a fetus for a genetic condition or used *in vitro* fertilization. Respondents were asked if they had ever heard of PGD; immediately after answering that question, a definition of PGD was provided. Awareness and knowledge of PGD was assessed with items such as awareness of PGD in general, awareness of PGD testing procedures, nurses’ educational needs about PGD were assessed with items that included acceptability of use of PGD among hereditary cancer families, factors influencing the decision to discuss PGD with patients (age, reproductive history, family history, *etc.*), concerns regarding PGD (cost, safety, religion, *etc.*), benefits of PGD, previous personal experience with PGD, barriers to PGD discussion with patients, and informational preferences regarding PGD.

### 2.3. Procedures

Nurses interested in the study filled out an online survey through a link contained in an email. All respondents could enter a raffle to receive one of four $25 gift cards for their participation. Surveys requests were e-mailed in April 2012 and the survey closed 31 May 2012.

### 2.4. Data Analysis

Completed surveys were transferred to an ACCESS datafile and analyzed by conducting basic descriptive statistics and bivariate analyses. *P* values were calculated using Chi Square tests with <0.05 as the level of significance. Statistical Package for the Social Sciences (SPSS) was used to conduct the analyses.

## 3. Results and Discussion

### 3.1. Participant Demographics

The survey link was mailed to 402 nurses on the listserv. Two hundred and one nurses (50%) completed the survey. The majority of respondents were female (*n* = 188), white (*n* = 175), catholic (*n* = 81) or atheist (*n* = 63), held a RN/BSN degree (*n* = 83), and provided outpatient care at the cancer center (*n* = 102). Respondents worked in a variety of clinic/cancer sites with the majority in breast (*n* = 12); followed by hematology (*n* = 9) and infusion center (*n* = 8). See Table 1.

**Table 1:** Demographics (*n* = 201) *.

Gender	Total
Male	13
Female	188
**Age**	
25 or under	4
26–35	33
36–45	47
46–55	64
56–65	44
66 or above	4
**Ethnicity**	
White	175
Black or African American	6
Asian	5
More than one race	6
Other	5
Prefer not to respond	4
**Education**	**Total**
BSN	83
Associates Degree	72
MSN	25
Diploma	14
Other	1
**Inpatient or Outpatient**	
Outpatient	102
Inpatient	77
**Clinic Type**	
Breast	12
Hematology	9
Infusion Center	8
Gastrointestinal	6
Gynecology	5
Radiation Oncology	5
Thoracic	4
Genitourinary	3
Neuro	2
Other	41
**Religion**	
Catholic	81
Atheist/Agnostic	63
Protestant (Baptist, Presbyterian, *etc.*)	8
Islamic	3
Prefer not to respond	23
Other	22

* Demographic variables differ in totals due to missing responses. The response rate for the survey was 50% (402 nurses on listserv).

### 3.2. Knowledge and Awareness

Sixty-four percent (*n* = 129) of respondents were aware of a genetic counseling program to which they could refer a cancer patient for more information about hereditary cancers. Six percent (*n* = 12) had previously personally had a cancer-related genetic test to identify potential mutations.

Seventy-eight percent (*n* = 156) of respondents had not heard of PGD prior to the survey. Among the 18% (*n* = 37) who had heard of PGD, 59% (*n* = 22) rated their knowledge as limited. Although not all respondents had heard of PGD prior to the survey, 29% (*n* = 58) said they would consider using PGD themselves.

Nurses who indicated that they were aware of PGD were mostly female (*p* = 0.276), not Hispanic or Latino (*p* = 0.123), Caucasian (*p* = 0.817), had an AA/Diploma (*p* = 0.191), were outpatient based (*p* = 0.310), 36 years and over (*p* = 0.417), Catholic (*p* = 0.017), had children (*p* = 0.806), or did not intend to have more children (*p* = 0.465). Table 2 shows the awareness of PGD categorized by gender, ethnicity, educational background, inpatient or outpatient based, clinic type, age, religion, have children, or want future children.

**Table 2:** Knowledge of PGD.

Prior to this survey, have you ever heard of preimplantation genetic diagnosis?				
		Aware	Not Aware	Total *
Gender (*p* = 0.276)	Male	1	12	13
	Female	36	144	180
	Total	37	156	**193**
Ethnicity (*p* = 0.123)	Hispanic or Latino	1	17	18
	Not Hispanic or Latino	36	139	175
	Total	37	156	193
Race (*p* = 0.817)	Caucasian	33	137	170
	Not Caucasian	4	19	23
	Total	37	156	**193**
Education (*p* = 0.191)	AA/Diploma	25	87	112
	BSN/MSN/PHD/Other	12	69	81
	Total	37	156	**193**
Inpatient or outpatient (*p* = 0.310)	Inpatient	12	59	71
	Outpatient	23	78	101
	Total	37	156	**193**
Clinic Type (*p* = 0.261)	Women’s Cancer (Breast, GYN)	5	12	17
	Others	32	144	176
	Total	37	156	**193**
Age (*p* = 0.417)	35 and under	5	30	35
	36 and over	32	126	158
	Total	37	156	**193**
Religion (*p* = 0.017)	Atheist/Agnostic	14	49	63
	Catholic	20	59	79
	Others	3	48	51
	Total	37	156	**193**
Have Children (*p* = 0.806)	Yes	28	121	149
	No	9	35	44
	Total	37	156	**193**
Future Children (*p* = 0.465)	Yes	4	23	27
	No	32	121	153
	Total	36	144	180 **

* Variables differ from overall total (*n* = 201) due to a missing response for the “awareness” item; ** Variable differs in total due to missing responses.

**Figure 1:**
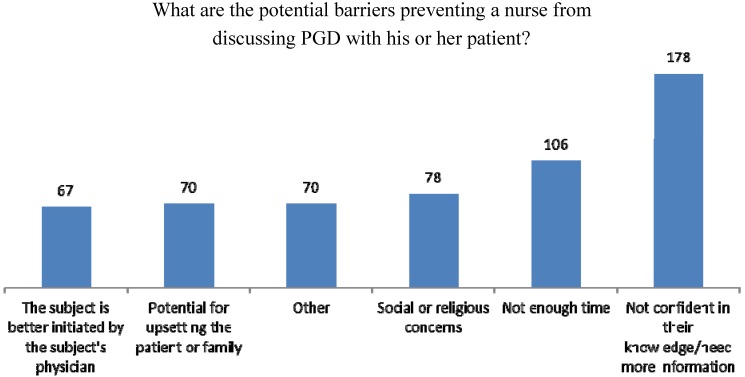
Barriers to PGD.

The majority, 89% (*n* = 178), said the barrier preventing them from discussing PGD with patients was a lack of confidence in their knowledge on the subject followed by 53% (*n* = 106) who reported not having enough time to talk about PGD, 39% (*n* = 78) who had social or religious concerns about discussing PGD with patients, 35% (*n* = 70) and 33% (*n* = 67) who believed that the topic was better initiated by a physician (Figure 1).

### 3.3. Educational Needs

Sixty-six percent (*n* = 133) thought PGD was an acceptable option for families with hereditary cancer syndromes. The same percentage thought people with a strong family history of breast or ovarian cancer may choose not to have children due to fear of passing on a gene mutation. Eighty-one percent (*n* = 163) thought individuals with a strong family or personal history should be provided with information about PGD.

When thinking about PGD, the majority of “worry” was focused on the ability of people to afford the technology, 40% (*n* = 80) followed by concerns that “the technology would be used for the wrong purpose, 22% (*n* = 45) and that using the technologies was “too much like playing God”, 16% (*n* = 33).

The majority, 53% (*n* = 106), would want more information about PGD from a genetic counselor followed by 21% (*n* = 42) who reported wanting more information about PGD from a physician with training in genetics.

## 4. Discussion

The present study aimed to identify knowledge and awareness of PGD and to determine which factors associated with awareness of PGD as well as educational needs of oncology nurses at the Cancer Center. Although PGD is a highly debated and controversial topic for many healthcare providers, over half of the nurses thought PGD was an acceptable option for families with hereditary cancer syndromes and thought individuals with a strong family or personal history should be provided with information about PGD. These attitudes are consistent with past studies that have found women who are at high-risk for hereditary cancer to be receptive to PGD and wish to learn more [16].

Similar to patients’ and families’ views on PGD and other healthcare providers in the field [29], nurses in this study expressed support for PGD for families with hereditary cancer syndromes but also expressed moral and ethical concerns about PGD such as the perception that use of PGD was like playing God and that it could be easily used for the wrong purposes. These concerns may serve as barriers preventing oncology nurses from obtaining more information about PGD and relaying this information to their patients. Consequently, efforts should be made to alleviate these fears and concerns so that nurses are well-informed about PGD and can provide empirically based information to their patients who can then make well-informed decisions.

Although more than half of the respondents were aware of a genetic counseling program to which they could refer a cancer patient for more information about hereditary cancers, a large percentage of participants reported that they had not heard of PGD prior to the survey or had limited knowledge, suggesting that promotion of PGD as an option for high risk families needs attention. Patients believe that having this knowledge remains their personal choice [19] and The American Society for Reproductive Medicine suggests that informing patients of this option is good ethical practice [33].

The lack of dissemination of PGD information may be explained by a barrier reported by the nurses, that is, their belief that PGD discussions were better initiated by the patient’s physician who may have greater knowledge on the topic than themselves. However, nurses play an important role in the dissemination of reproductive options. Given this, patient and family decisions regarding PGD are highly influenced by how much and what information is presented to them by healthcare professionals. Discussion barriers may be best alleviated by educating oncology nurses about PGD and for practice settings to provide clear direction and delineated roles regarding PGD discussions with high risk families. 

As the field of oncology related genetics is growing at an exponential rate, the availability of trained professionals is lagging [34]. Several studies have concluded that nurses benefit from targeted oncology genetics education by improving confidence in discussions with patients and understanding of patient treatment plans [35,36]. Further, it has been identified that nursing competencies and curriculum do not uniformly address cancer genetics [37,38]. While minimum competencies have been suggested by several groups [39,40,41] and training is available through a variety of modes and sources, limited research suggests there has not been widespread dissemination of this training. The National Human Genome Research Institute has developed “Essential Nursing Competencies and Curricula Guidelines for Genetics and Genomics” [42] stating the driving force behind the guidelines is an awareness that “genomics is a central science for nursing practice because essentially all diseases and conditions have a genetic or genomic component, options for care for all persons will increasingly include genetic and genomic information along the pathways of prevention, screening, diagnostics, prognostics, selection of treatment and monitoring of treatment effectiveness. These competencies are not intended to replace or recreate existing practice standards but are intended to incorporate the genetic and genomic perspective into all nursing education and practice. The goal is to have a basis by which to prepare the nursing workforce to deliver competent genetic and genomic focused nursing care”. Table 3 lists the known cancer genetic syndromes and their associated interventions [43]. Patients of childbearing age with any of these genetic syndromes may benefit from and be interested in PGD and nurses with training in cancer genetics may be an ideal first source to initiate discussions about childbearing goals, options, and referrals.

**Table 3:** Genetic syndromes and interventions [43].

Syndrome Name	Genes Responsible	Major Tumors/Cancers	Interventions
Cowden Syndrome	*PTEN*	Breast, thyroid, uterine; other benign tumors of various organs	Increased screening for all cancer/tumor types ^a^
Familial Adenomatous Polyposis and MutYH-Associated Polyposis	*APC*, *MutYH*	Colon cancer and polyps; small intestinal cancer	Prophylactic removal of colon, increased screening for other cancers
Familial Paraganglioma Syndrome	*SDHB*, *SDHC*, *SDHD*	Paragangliomas of head, neck, and abdomen	Increased screening for paragangliomas
Hereditary Breast-Ovarian Cancer Syndrome	*BRCA1*, *BRCA2*	Breast and ovarian cancer; prostate cancer in males; some other cancers slightly elevated	Increased breast screening, chemoprevention, and/or mastectomy; prophylactic removal of ovaries/fallopian tubes
Lynch Syndrome	*MLH1*, *MSH2*, *MSH6*, *PMS2*	Colon and uterine cancer; other cancers such as stomach, ovarian, urinary tract	Increased colon cancer screening and chemoprevention; prophylactic removal of uterus/ovaries; screening for other cancers
Juvenile Polyposis Syndrome	*MADH4* (aka *SMAD4*), *BMPR1A*	Gastrointestinal cancers, benign colon polyps	Increased screening for colon and small intestinal cancers
Li-Fraumeni Syndrome	*TP53*	Breast, brain, lung cancer, leukemia	Increased screening for breast cancer; consider research-based imaging for other cancers
Multiple Endocrine Neoplasia type 1	*MEN1*	Pancreatic cancer (neuroendocrine); pituitary and parathyroid tumors	Increased screening for pancreatic and other tumors
Multiple Endocrine Neoplasia type 2	*RET*	Medullary thyroid cancer; adrenal and parathyroid tumors	Prophylactic removal of thyroid; increased screening for other tumors
Peutz-Jeghers Syndrome	*LKB1* (aka *STK11*)	Gastrointestinal, breast cancer	Increased screening for colon, small intestinal, and breast cancer
Von Hippel-Lindau Syndrome	*VHL*	Renal cell carcinoma, brain cancer (hemangioblastoma), other benign tumors	Increased screening for all cancers/tumors

^a^ Screening refers to imaging (e.g., CT scan, PET scan, MRI), endoscopy (e.g., colonoscopy, upper endoscopy), and biochemical testing (e.g., blood and urine testing), or a combination of all of these screening recommendations for each syndrome are tailored to the type of cancer and the degree of risk.

## 5. Limitations

This study is not without limitations. The majority of the participants were female, self-identified as Caucasian, and were provided outpatient care at a single institution. Results may not be generalizable to all oncology nurses. Moreover, the response rate was 50%. Although this is in line with the response rate of other surveys posed to health care workers, the respondents might have been motivated to complete the survey for a reason that could bias the results.

## 6. Conclusions

This study supports previous findings that PGD is generally accepted among oncology healthcare providers but similar to patients and families who struggle with making this decision, nurses in this survey had limited knowledge and some reservations about the use of the procedures. It is essential that oncology nurses are educated about PGD so they can provide this information to patients and families who are interested in knowing their options. This includes not only providing education on the topics but also training on how to present this information in an unbiased and ethical manner. 
